# Smart Textile Display with Addressable Quantum Dot Light-Emitting Diode Based on Durable Ultrathin Metal Electrode

**DOI:** 10.1021/acsanm.5c00068

**Published:** 2025-03-18

**Authors:** Jiajie Yang, Jeong-Wan Jo, Yoonwoo Kim, Sung-Min Jung, Sanghyo Lee, Jong Min Kim

**Affiliations:** † Electrical Engineering Division, Department of Engineering, University of Cambridge, Cambridge CB3 0FA, U.K.; ‡ School of Materials Science and Engineering, Kumoh National Institute of Technology, Gumi 39177, South Korea

**Keywords:** quantum dot light emitting diode (QD-LED), smart textile display, flexible transparent electrode, mechanical durability, lateral driving architecture

## Abstract

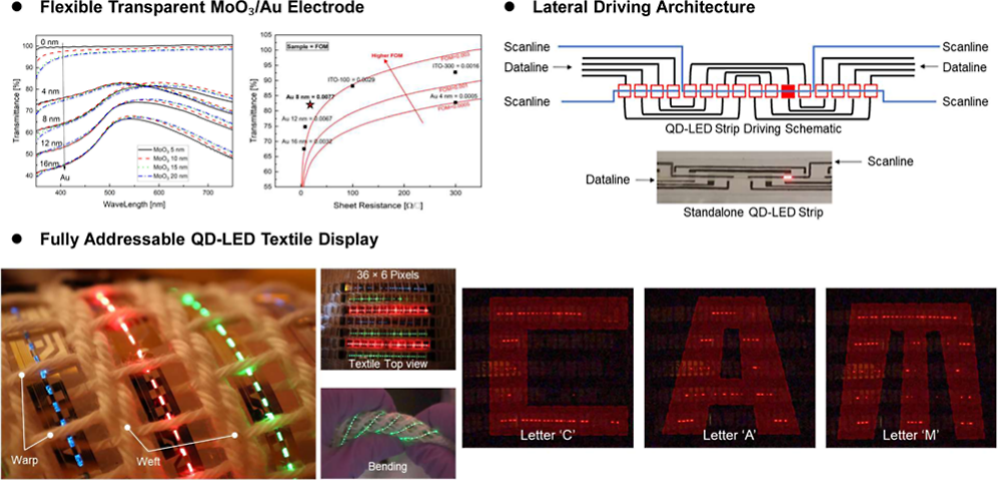

We report a fully addressable smart textile display using quantum dot light-emitting diodes (QD-LEDs) featuring a highly durable electrode. The key innovation lies in the development of an ultrathin oxide/metal hybrid structure for the durable electrode, which achieves mechanical bending durability surpassing the indium tin oxide (ITO) electrode. The optimized electrode, composed of MoO_3_ and Au, exhibits a transmittance of *T*_550_ = 81%, while maintaining a sheet resistance of *R*_s_ = 17.92, achieving a Figure of Merit (FoM) of 0.0077. The bending tests further demonstrate that the QD-LEDs with this electrode retain their luminance up to 6325 cd m^–2^ after undergoing 500 bending cycles at a bending radius of 5 mm. Furthermore, this study introduces not only a highly mechanically robust device, but also an integration method for textile systems by employing an innovative lateral driving display system architecture enabling the precise addressing of individual QD-LEDs in a textile display.

## Introduction

A smart textile is a form-factor-free electronic system, commonly referred to as electronic textiles (e-textiles), that has drawn considerable attention from researchers in recent years.^1^ It extends the functionalities of ordinary textiles to include intelligent functions and overcomes the physical shape constraints of conventional silicon wafer-based electronics. It does not seek to supplant traditional electronics but rather to forge a new realm. Over time, diverse textile functionalities have emerged,^2,3^ encompassing conductive properties,^4,5^ energy storage,^6^ and energy harvesting^7^ capabilities. Consequently, smart textiles hold vast potential across numerous sectors of future electronics, including nonwearable applications such as IoTs and next generation smart homes,^8−10^ as well as wearable applications such as smart clothing^11−13^ and healthcare.^14−16^

The field of smart textiles presents several distinctive challenges that need to be addressed, primarily due to the inherent properties of textiles compared to conventional planar electronics. The mechanical durability of textile-based devices is one of the most crucial parameters to be improved. Unlike conventional rigid electronics with a silicon substrate, smart textiles offer high freedom of form-factor, which means they experience diverse deformations during their typical operation conditions, requiring flexibility, bendability, rollability, and foldability. Among the various applications in smart textile electronics, textile displays are particularly compelling, as they not only serve as a platform for visual communication but also offer a high degree of aesthetic design freedom.^10,17−20^ To meet these demands of textile displays, high-performance transparent conductive electrodes (TCEs) combining high conductivity, optical transparency, and robust mechanical durability are essential. Indium tin oxide (ITO) is a well-established and highly transparent conductive oxide widely used as a TCE in optoelectronic devices such as flat panel displays, touch screens, solar cells, thin-film transistors, and LEDs due to its excellent transmittance and conductivity.^21−23^ However, its intrinsic brittleness, high production costs due to limited indium resources, and low work function present significant challenges for textile displays.^24−26^ As a result, various alternatives to ITO, including graphene, carbon nanotubes, metal nanowires, conducting polymers, ultrathin metal films, and metal grids, have been extensively studied.^27−32^ Oxide/metal/oxide (OMO) structured electrodes have emerged as promising candidates among alternative TCEs, as they utilized ultrathin metal films for high conductivity while oxide layers enhance optical transmittance and minimize reflection through interference effects. Notably, molybdenum trioxide (MoO_3_)/gold (Au)/MoO_3_ structures stand out due to the deep work function of MoO_3_ and Au for efficient hole injection, as well as the ductility of Au, which enhances flexibility, making them highly suitable for smart textile displays.^33−39^

In addition to these scientific challenges associated with high freedom of form-factor, large-scale manufacturing is also an important factor to consider. Although, some studies^40−42^ have shown intriguing designs for functional smart textiles, their manufacturability remains confined to laboratory settings, lacking scalability. For instance, alternating current electroluminescent is one of the most popular technologies employed by many textile displays. However, it requires a high level of alternating voltage, which necessitates a complex driving circuit. Such an intrinsic limitation hinders its application in futuristic high-definition, high-refresh-rate displays. Therefore, direct-current-driven light-emitting diode (LED) displays are more promising and have more potential in e-textile display applications. Quantum dots (QDs) have emerged as cutting-edge emissive materials for next-generation displays,^2,12,43−45^ offering exceptional color purity and efficiency, making them highly promising for direct-current-driven LED display applications. However, to the best of our knowledge, there have been few demonstrations of QD-LED textile displays that simultaneously achieve high mechanical stability and scalability for mass production. To enable the integration of QD display technology into fiber/textile electronics and ensure both large-scale manufacturability and commercial viability, it is crucial to develop a new, simplified driving method for textile QD displays that also secures mechanical flexibility.

Here, the development of a fully addressable QD-LED textile display is presented, focusing on the study of flexible electronic devices for smart textiles at all levels from the material up to system integration. This report includes the study of fundamental aspects of flexible transparent electrodes, highly robust flexible QD-LEDs, and fiber-based integration/interconnection technology. Unlike other reports^17,20^ that employ a criss-cross driving pattern for interconnection, this work utilizes a lateral architecture. The lateral architecture drives and addresses each individual QD-LED pixel from both ends, eliminating the need for a vertical connection. Each QD-LED strip is a self-contained standalone subsystem which can be addressed separately. This innovative lateral driving architecture significantly enhances the stability and mechanical robustness of the textile display system. The system features an ultrathin, flexible, and transparent metal electrode composed of MoO_3_ and Au, combined with a novel lateral driving mechanism. Together, these advancements enable a mechanically durable and fully addressable QD-LED textile display that is scalable to large areas.

## Experimental Section

### Fabrication of Flexible Transparent Electrode

The fabrication of the ultrathin MoO_3_ and Au metal electrode starts with the cleaning of the substrate. The polyethylene terephthalate (PET) substrate, purchased from Sigma-Aldrich, is first placed in acetone and sonicated for 10 min using the Fisher Scientific FB15051 sonicator. It is then sonicated in IPA for an additional 10 min and dried with an N_2_ blow gun. For O_2_ plasma treatment, the substrate is placed in the chamber under vacuum, and the O_2_ plasma treatment is performed for 5 min. The cleaned substrate is then transferred to the thermal evaporator in the glovebox. An MoO_3_ layer is thermally deposited with thicknesses of 5, 10, 15, or 20 nm. Subsequently, a layer of Au is deposited immediately without removing the sample from the evaporator, with thicknesses of 0, 4, 8, 12, or 16 nm. Therefore, a total of 20 MoO_3_/Au (MA) electrode samples are prepared. The evaporation materials, including molybdenum oxide, gold (G4-5005-M), silver (S5-5005-M), and aluminum (A1-5007-M), are all purchased from Testbourne Ltd., UK.

### Evaluation of Flexible Transparent Property

Properties including transmittance, sheet resistance, and mechanical durability are examined and evaluated. The transmittance was measured using a UV–vis Cary 7000 spectrometer. The transmittance of the clean PET substrate was first measured and set as the baseline. The sheet resistance was measured using the four-probe measurement technique through a Jandel four-probe system and a Keithley 6430 Source Meter. The cylindrical probe head attached to the Jandel four-probe system has four tungsten carbide probes. The probes have a diameter of 0.4 mm and are spaced at a distance of 1.00 ± 0.01 mm. The mechanical stability is assessed using a bending machine constructed in-house, paired with a Keithley 2604b Source Meter, which measures the resistance change over 6000 cycles of bending at a specific radius of curvature. Each sample has a substrate size of 50 mm × 15 mm × 125 μm (length-width-thickness) and is compressed to a desired radius of curvature (*R* = 5 mm) in 0.5 s and then released to flat in 0.5 s (Figure S1). The real-time measurement of the resistance is recorded at a rate of 40 Hz (25 ms per measurement). Both the tension bending test and the compression bending test are carried out for each material. The difference between the two types of tests is illustrated in Figure S2. Two commercially available PET/ITO substrates, both purchased from Sigma-Aldrich, with a nominal sheet resistance of 100 Ω sq^–1^ (ITO_100) and 300 Ω sq^–1^ (ITO_300), were also included in the measurement, along with other thermally evaporated metal electrodes (Ag, Au, and Al). The ITO, Al, Ag, and MA samples have initial resistances of 1576.95, 1.41, 0.74, and 2.01 Ω, respectively.

### Fabrication of QD-LED Devices

The fabrication of the QD-LED starts with the preparation of the solutions. For the reduced phosphomolybdic acid (PMA-r) solution, 45 mg of phosphomolybdic acid (PMA) was first dissolved in 3 mL of acetonitrile in a glass vial and then baked on a hot plate at 200 °C overnight. Note that the entire process was carried out in a glovebox to allow dehydration and reduction to take place. The poly(9,9-dioctylfluorene-*alt*-*N*-(4-*s*-butylphenyl)-diphenylamine) (TFB) was dissolved in chlorobenzene at a concentration of 10 mg mL^–1^. The solution was magnetically stirred overnight to achieve a homogeneous solution. The CdSe/ZnS red, green, and blue QDs were purchased from Suzhou Xingshuo Nano. The original solution (25 mg mL^–1^ in octane) was diluted to 12.5 mg mL^–1^ with octane. The magnesium doped zinc oxide (MZO) in butanol was made in-house and has a concentration of 25 mg mL^–1^, with 15% Mg doping. For the ultrathin metal electrode of the MA QD-LED, the MA electrode was first fabricated using the method described previously. The PMA-r solution, TFB, QD, and MZO layers were spin-coated and baked under specific conditions. The spin speed was set to 4000 rpm for the PMA-r solution, TFB, and QD layers, and 2000 rpm for the MZO layer, all with a spin time of 30 s. The baking temperatures were 100, 150, 100, and 100 °C, respectively, with a uniform baking time of 10 min for all layers. For ITO electrode-based samples, only the MA electrode is replaced with a commercial ITO substrate; the rest of the procedures remain unchanged. The bending durability is quantified in terms of luminance, current density, and EQE changes over 500 bending cycles. Similar to the flexible transparent electrode (FTE) mechanical durability test, the QD-LEDs are loaded on the bending machine and bent to a radius of 5 mm. Each cycle consists of a 0.5 s bending and 0.5 s releasing section. As it is impractical to measure the luminance in real-time, the luminance and other electrical properties (current density, EQE) were measured every 100 cycles, specifically at 0, 100, 200, 300, 400, and 500 cycles. The Hamamatsu PMA-12 multichannel photonic analyzer, along with the Keithley 2400 Source Meter, was used to capture optoelectronic data, including EQE, current efficiency, luminance, and spectral intensity from wavelengths of 350 to 1100 nm.

### Fabrication of Textile QD-LED Display

The substrate was first cleaned using the steps described in the previous section. The first resist layer, LOR10B, was coated using spin coating with a two-step spin profile. The first step was 500 rpm for 10 s, and the second step was 3000 rpm for 45 s. This first step was to spread the resist evenly on the substrate, while the second step set a final film thickness of ∼1 μm. The first layer was baked at 150 °C for 5 min. Then, the second layer, AZ5214E, was spin-coated using the same speed profile. It was baked at 110 °C for 1 min. The prepared substrate was then exposed to 120 mJ cm^–2^ using an MABA6 356 nm UV light source. The photomask is a Laser Photoplot Film mask custom-made by JD PHOTO DATA and has a resolution of 10k DPI. The exposed substrate was soaked in AZ726MIF developer for 90 s. This optimized developing time ensures that a desirable undercut profile can be formed. The developed substrate was rinsed under DI water for 60 s and then dried using an N_2_ gun. After the deposition of MoO_3_/Au, the sample was soaked in Remover PG for 30 min for lift-off. Note that during this process, the temperature was maintained at room temperature (20 °C) since the adhesion of the MA electrode degrades at elevated temperatures. The substrate was then rinsed with acetone to remove the residual Remover PG. The QD-LED fabrication process is the same as mentioned previously. After production, the fabricated fiber LEDs were cut into fibers and were ready for weaving. The textile was woven using the manual loom in a plain weave pattern. The yarn used is a double knit (DK) 100% cotton by Patons, rated for machine washability. The shifter registers used are 74HC595 TSSOP surface-mount IC chips (MC74HC595ADTR2G). It is an 8-bit serial-in and parallel-out shifter with an operating voltage range of 2 to 6 V. Only 6 bits are used since the fibers have 6 pins on one end. The final textile display has an overall size of 75 mm × 78 mm, with 36 × 7 pixels.

## Results and Discussion

**Figure 1:**
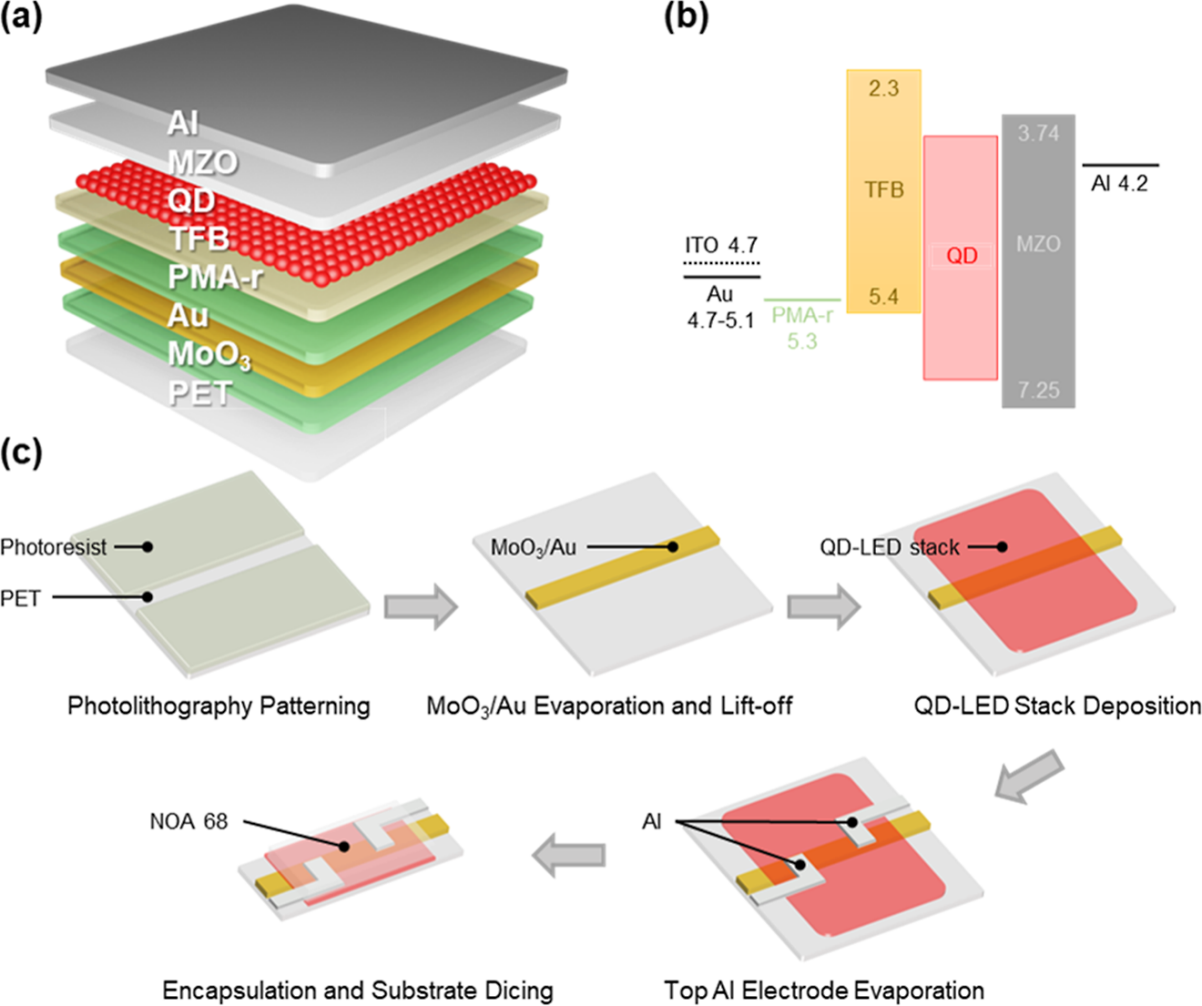
(a) Structure of the QD-LED device, comprising a PET substrate, a MoO_3_/Au flexible transparent electrode, a PMA-r hole injection layer, a TFB hole transport layer, a QD light emission layer, a MZO electron transport layer, and an Al top electrode. (b) Band diagram of the QD-LED device. (c) Schematic illustration of the fabrication process for the QD-LED strip device, designed for integration into the textile display.

Figure 1 shows the schematic diagram of the proposed device structure, the band diagram, and fabrication process. A conventional bottom emitting structure is employed in this study. It consists of a polyethylene terephthalate (PET) substrate, a MoO_3_/Au flexible transparent electrode, a reduced phosphomolybdic acid (PMA-r) hole injection layer, a poly(9,9-dioctylfluorene-*alt*-*N*-(4-*s*-butylphenyl)-diphenylamine) (TFB) hole transport layer, a QD light emission layer, a magnesium doped zinc oxide (MZO) electron transport layer, and an Al top electrode. The developed ultrathin metal MA electrode is employed in QD-LED device to examine its device performance and compare it with the standard ITO electrode. The hole injection layer (HIL) is a crucial component of the QD-LED. The purpose of the HIL is, as the name suggests, to aid the injection of holes from the anode to the QD and block the movement of electrons from the anode to the QD. Although PEDOT:PSS has been widely used as HIL material for QD-LEDs, it has a challenge of poor wetting on the Au metal surface due to the high surface energy of Au. Moreover, with prolonged exposure (>10 s) to PEDOT:PSS, the ultrathin metal MA electrode can be easily damaged by the water solvent of PEDOT:PSS.^36,39^ Thus, PMA-r was employed as an alternative HIL material in this study because it is free from water solvent-induced damage and facilitates efficient hole injection owing to its deep work function.^33−38^ The PMA-r HIL was prepared by dehydration and reduction of PMA, as described in the experimental section. In this structure, the active QD-LED pixel area is defined by the Al metal top electrode deposition, as the Al electrode physically determines the regions where charge injection occurs (Figure 1c).

**Figure 2:**
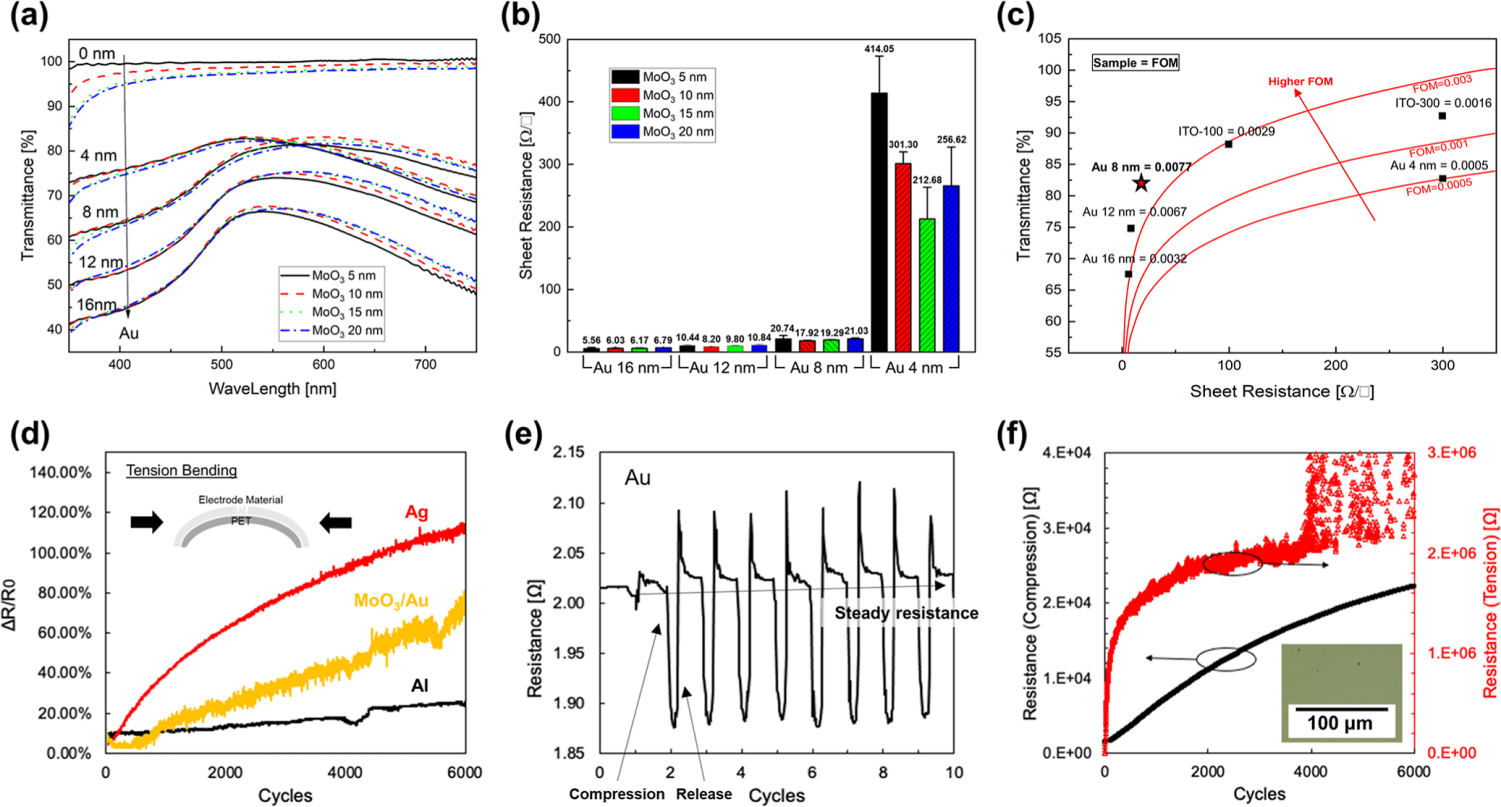
(a) Transmittance and (b) sheet resistance of the MoO_3_/Au electrode layers with various thickness combinations. (c) FoM values of all samples, with the Au 8 nm sample showing the highest FoM of 0.0077. (d) Resistance changes over 6000 cycles of bending to a 5 mm radius of curvature for Ag, MoO_3_/Au, and Al electrodes. (e) Resistance changes during the first 10 cycles of bending, highlighting a steady increasing trend. (f) Resistance changes over 6000 cycles of bending to a 5 mm radius of curvature for the ITO electrode.

Flexible transparent electrode (FTE) is the key component to realize a robust and mechanically durable flexible QD-LED. The ultrathin oxide/metal electrodes are fabricated using conventional vacuum deposition to ensure their performance, stability, and mass production compatibility. For MoO_3_-only samples, all four samples (5, 10, 15 and 20 nm) have transmittance over 95% at the visible spectrum (400–800 nm) (Figure 2a, Au-0 nm group). When coated with 4 nm Au, the transmittance drops below 85%, and the thicker the Au layer, the lower the transmittance. It is noteworthy that the transmittance of 8 nm Au samples at a wavelength over 550 nm is higher than that of the 4 nm Au samples, which does not follow the common trend, regardless of the MoO_3_ thickness. All MoO_3_/Au (MA) samples exhibit concave-shaped transmittance curves, with the highest transmittance falling under the visible range from 500 to 600 nm. This indicates such MA electrodes are strong candidates for display or lighting applications, such as QD-LEDs. To examine the correlation between transmittance variation and MoO_3_ thickness in relation to color coordinates, we measured the electroluminescent (EL) spectra of QD-LED devices with MA electrodes. Notably, all QD-LED devices exhibited a consistent peak wavelength of 629 nm and a full width at half-maximum (FWHM) of 25 nm, regardless of MoO_3_ thickness (Figure S3). This result is consistent with previous findings, which indicate that microcavity effects that typically cause shifts in wavelength or color coordinates are negligible due to the high transparency of MA electrodes.^36,46^ These findings suggest that despite variations in transmittance, MA electrodes remain strong candidates for QD-LED display and lighting applications. The simulated transmittance over the visible spectrum ranging from 380 to 780 nm for the various thickness configurations of the MA electrodes identical to the experimental conditions are plotted in Figure S4 for theoretical comparison. As the Au layer thickness increases, the transmittance decreases, with less reduction observed in the green region, due to the enhanced reflection from the thicker Au layer and the increasing extinction coefficient of the Au layer beyond 500 nm. Conversely, increasing the thickness of the MoO_3_ layer leads to higher transmittance due to interference effects within the MoO_3_ layer. In general, a MoO_3_ thickness of over 10 nm (10, 15 and 20 nm) does help further improve the transmittance, but in a very limited way, and only applicable to wavelengths over 550 nm. Such effects are minimal at wavelengths below 550 nm. No clear indication suggests that 20 nm thick MoO_3_ would greatly improve the transmittance. The results in Figure S5 show the T_550_ of ITO_300 and ITO_100 is 91% and 87%, respectively, with the highest transmittance over 90% at the infrared region (longer wavelength 750 nm), following the commonly agreed trend,^33,47,48^ where thicker films typically exhibit lower transmittance.

In terms of sheet resistance, MA samples with 4 nm Au thickness have a particularly high sheet resistance of over 200 Ω sq^–1^, indicating the lack of formation of a continuous Au thin film. The other samples all show a sheet resistance below 22 Ω sq^–1^, proving their viability as an FTE. These results are shown in Figure 2b, which highlights the relationship between Au thickness and sheet resistance for the MA samples. While the reduction in sheet resistance is primarily attributed to the increase in Au thickness, the presence of MoO_3_ also plays a crucial role beyond its optical benefits. Specifically, underlying MoO_3_ acts as a seed layer, promoting the formation of a more continuous Au film even at ultrathin thicknesses. This improved film continuity facilitates electron percolation, thereby reducing sheet resistance despite the ultrathin nature of the Au layer.^33−35,37,38^ A more meaningful value is the FoM is calculated as ΦH = *T*(10)/*R*_s_,^49^ where *T* and *R*_s_ are the transmittance and sheet resistance, respectively (Figure 2c). The 8 nm Au has the best FoM of 0.0077 among all the samples. This value is more than twice the benchmark of commercially available ITO electrodes. The transmittance and sheet resistance of all samples are summarized in Table S1. The developed MA electrode with 10 nm of MoO_3_ and 8 nm of Au is the most suitable combination for QD-LED applications.

In bending durability measurements (Figure 2d,e), all metal electrode materials showed excellent bending durability, attributed to their inherent ductile and flexible nature. Among them, the maximum resistance change after 6000 cycles of bending was below 120%, observed for Ag under tension bending. However, it increased only from 0.74 to 1.63 Ω, which remains an excellent conductor. While these results indicate that Ag, as employed in the MoO_3_/Ag/MoO_3_ electrode,^50−52^ is a promising conductor, its long-term stability is compromised by oxidation under the acidic conditions of PEDOT:PSS and PMA-r solutions.^53,54^ In contrast, despite the higher cost and rarity of Au, its deep work function, superior ductility and enhanced oxidation resistance make it essential for achieving reliable performance and long-term stability in highly conductive transparent electrodes.^33−39^ For MoO_3_/Au, the changes in tension and compression show a similar trend, with maximum changes of 80%. The resistance changes during the first 10 cycles (compression) of Au are presented in Figure 2e, revealing insights into resistance changes per bending cycle. Initially, resistance decreases due to the shorter conductive path after compression. Subsequently, the resistance slightly increases beyond its original value. Notably, the resistance change during the first cycle is significantly smaller than those in subsequent cycles. This may be attributed to metal work hardening. Before bending, the metal thin film is relatively soft and ductile, resulting in minimal changes during the first cycle. After work hardening, the film becomes more vulnerable to cracks, leading to greater resistance changes from the second cycle onward. Nevertheless, the steadily increasing trend is of greater interest, and this increase is significantly lower than that observed in ITO.

On the other hand, ITO exhibits greater susceptibility to bending in both compression and tension (Figure 2f). The result agrees with the findings reported in other studies.^55−57^ The degradation is particularly pronounced under tension. This could result from crack propagation and exfoliation of the ITO. Under tension, ITO cracks, and the crack widens as the bending radius decreases, leading to increased resistance. This explains the sharp increase in resistance during the first 2000 cycles. After more cycles, ITO delaminates and exfoliates from the substrate, causing it to fail as an electrode after 4000 cycles. Conversely, ITO under compression shows a much more stable change in resistance, remaining conductive with a resistance of 20,000 Ω after 6000 cycles.

**Figure 3:**
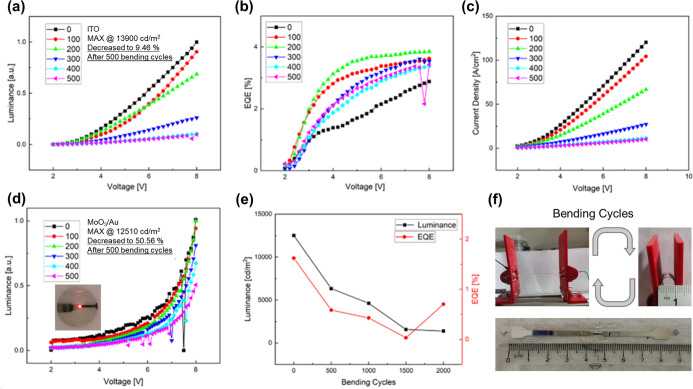
(a) Luminance, (b) EQE, and (c) current density curves as a function of the applied voltage for the ITO-based QD-LED device over 500 bending cycles. The ITO-based QD-LED device is vulnerable to bending, and its luminance drops to 9.46% of the initial luminance. (d) Luminance curves as a function of the applied voltage for the MoO_3_/Au-based QD-LED device over 500 bending cycles. The MoO_3_/Au-based QD-LED device shows a luminance drop to only 50.56% after 500 cycles. (e) Luminance and EQE trends of the MoO_3_/Au-based QD-LED device over 2000 bending cycles. (f) Optical image of the QD-LED device mounted on the bending test machine.

Figure 3 shows the bending test results of QD-LEDs with ITO and MA electrodes. In general, a decreasing trend in luminance and EQE is expected as the number of bending cycles increases. For the ITO-based device, the luminance drops sharply, retaining only 9.46% of its initial value after 500 bending cycles (Figure 3a–c), confirming its limited durability. In contrast, the QD-LED device with a pristine MA electrode demonstrates better durability, as shown in Figure 3d. After 300 cycles, the luminance decreases significantly to 10,258 cd m^–2^, which is still 82% of the original maximum luminance of 12,510 cd m^–2^ at 8 V. As the bending cycle increases further to 500 cycles, the luminance drops to 50.56% of its initial value. It should be noted that although our devices are fabricated on PET that is processed into a fiber form factor for textile integration, the initial performance of the QD-LED device with the MA electrode is comparable to that of flexible QD-LEDs on PET employing similar OMO electrode structures.^33,34^ Moreover, our devices exhibit superior electro-optical characteristics compared to other textile LED devices fabricated on similar PET fiber substrates.^17−19,58^ However, further process optimization and structural improvements are required to achieve the electrical performance of state-of-the-art flexible QD-LEDs.^2,12^

To further assess the maximum durability and long-term performance of the MA QD-LED, additional bending tests up to 2000 cycles were conducted, and the trend is presented in Figure 3e. Although the MA electrodes were found to be electrically stable up to 6000 cycles (Figure 2d), a sharp decrease in luminance is observed in the MA QD-LED after only 2000 cycles, suggesting that factors beyond the electrode contribute to luminance degradation. First, delamination of the encapsulation in the QD-LED strip was clearly observed. This delamination typically starts at the edges of the strip and propagates toward the center. When multiple layers are stacked, delamination occurs at the interface with the weakest interlayer adhesion force. Although the Norland 68 encapsulation exhibits strong adhesion to the Al and clean PET substrate, the Al top electrode detaches easily from the underlying layers, causing catastrophic damage to the QD-LED device.^59,60^ Second, after 1000 cycles of bending, the PET substrate itself exhibits permanent plastic deformation (Figure S6), which may further contribute to luminance degradation. To further investigate the underlying causes of luminance degradation, we conducted additional bending tests (up to 2000 cycles) on the PMA r, QD, and MZO layers to evaluate their mechanical stability (Figure S7). The results showed that no visible cracks appeared in these layers, which is consistent with the fact that ultrathin films are generally less susceptible to bending-induced damage.^61,62^ This result implies that direct mechanical failure is not the primary cause of luminance degradation. To address these delamination and degradation issues, future research should focus on introducing neutral layer structures and developing new designs to enhance the flexibility of QD-LED strips.^22,62−64^ In addition to the bending durability test, a lifetime measurement of QD-LED was also performed. The results indicate that the lifetime of the MA QD-LED device reaches 5 h for *T*_90_ and 887 h for *T*_75_ (Figure S8). This lifetime is beneficial for realizing a stable textile display system.

**Figure 4:**
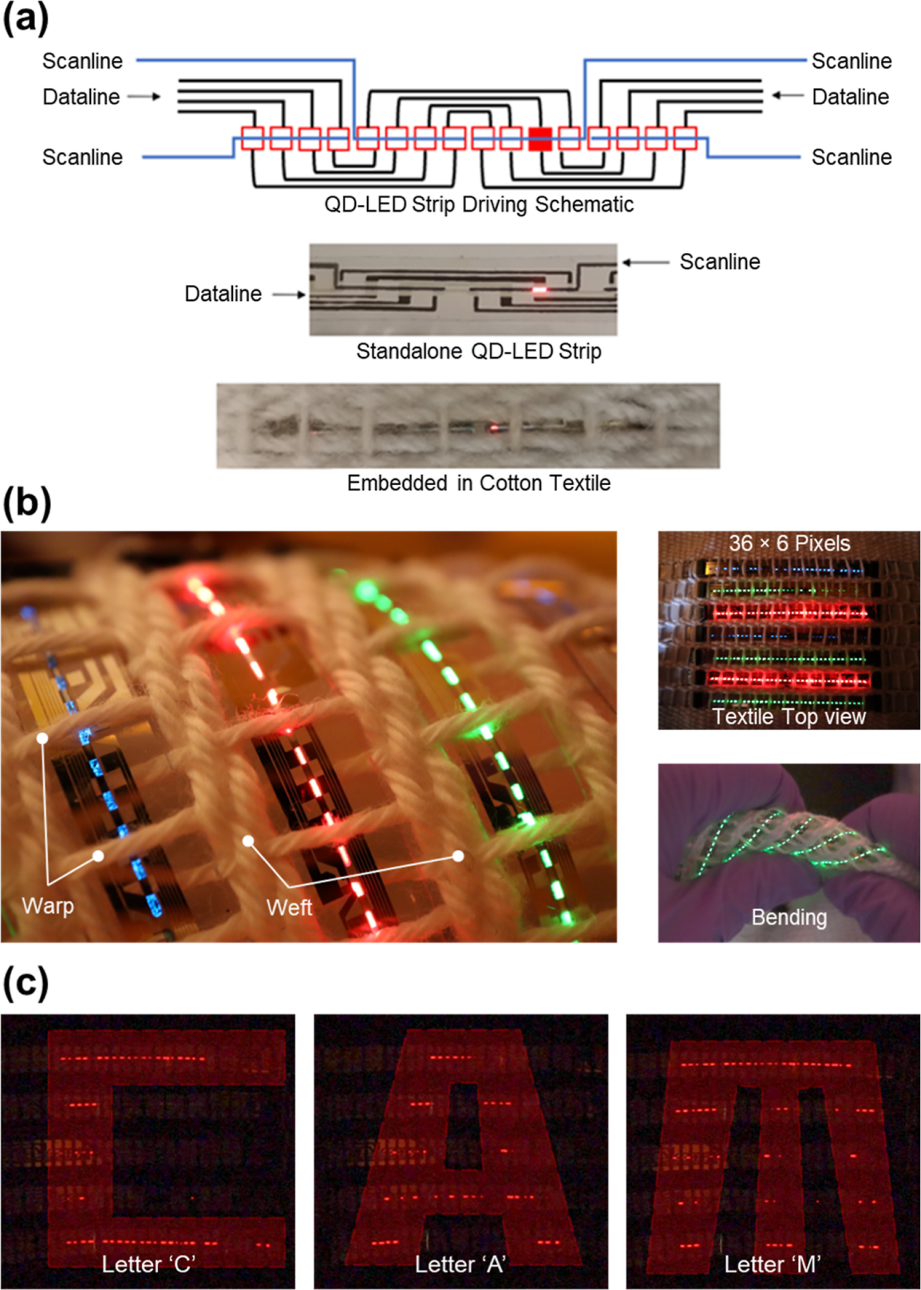
(a) The driving schematic of the addressable QD-LED strips, showing the scan and data lines. (b) The fabricated 36 × 6 pixels full-color QD-LED textile display under bending, demonstrating its flexibility. (c) Demonstrating the addressability of the QD-LED textile display, displaying the letters “C”, “A”, and “M”.

The novel lateral driving architecture addresses the challenging interconnection problem in the fabrication of high-resolution textile displays (Figure 4). This electrode architecture is compatible with a fiber form factor, eliminating the need for vertical connections, with each fiber functioning as a standalone addressable device. Furthermore, the fabrication process is compatible with existing manufacturing technologies, offering the potential for large-area scalability and high-resolution. The design concept is illustrated in Figure 4a by a 16-pixel strip. The 4 data lines run from the left of the strip to the right of the strip. The scan lines are attached to the QD-LEDs, and the leads run to one of the ends depending on the position. Each pixel can be driven individually through multiplexing.

Photolithography is more suitable for high-resolution applications over the shadow mask and has potential to further increase pixel density. Here, it is used for a 36-pixel fiber (100 mm × 5 mm × 125 μm, 6 groups × 6 pixels, 0.5 mm × 1.0 mm pixel size). It was discovered that when an etching process was used after photolithography, the MA electrode detaches from the substrate when developed using the TMAH-based developer, thereby getting damaged and becoming unusable. Therefore, the lift-off technique is used to pattern the electrode (Figure S9). In this research, double-layer photolithography was used to realize the undercut structure in a more controllable manner compared to single-layer photolithography. PET was chosen as the substrate because of its high compatibility with photolithographic processes, which have been demonstrated for various fiber forms in the literature.^17−19,58^ This compatibility allows for precise control over electrode and pixel dimensions, which is essential for high-resolution applications. Although our QD-LEDs are fabricated on PET using a spin-coating method, we refer to them as textile QD-LED displays because their strip-type configuration, with a high aspect ratio greater than 30, mimics PET-based fibers that can be easily woven into fabrics.^65,66^ Our design, which features a high aspect ratio within the range of a few millimeters or submillimeters, qualifies the devices as fiber-like and suitable for integration into textile systems.

Finally, the 36 × 6 pixel full-color QD-LED textile display under bending, demonstrating its flexibility, is shown in Figure 4b. Displaying the letters “C”, “A”, and “M” demonstrates the addressability of the QD-LED textile display (Figure 4c). At each end of the fiber, there is a custom-designed printed circuit board (PCB), shown in Figure S10. The PCB contains a shift register to complete the serial-to-parallel processing of the signal. The serial signal is produced by a microcontroller and is sent out to the first shift register via a 6-core IDC ribbon cable (Figure S11). The microcontroller is a commonly available Arduino Uno loaded with custom code. While driving, the shift register is latched on, and the serial data is transmitted to the shift register. Once it receives the data, the latch pin is grounded. In every cycle, one group of 6 pixels is turned on for multiplexing, and the cycle repeats 6 times to complete the entire frame. All the shift registers are daisy-chained so that the serial data is passed on to the additional shift registers in sequential order. Note that the order of the pixels in the adjacent groups is reversed, and the data is also reversed accordingly. In this work, as a proof of concept, we have implemented a low-resolution QD-LED textile display based on a lateral driving architecture using an Arduino Uno microcontroller. However, our fabrication approach, which uses photolithography to achieve an electrode architecture compatible with fiber form factors, is sufficient to fabricate fine-patterned interconnections for implementing high-resolution QD-LED textile displays. By integrating display driver ICs commonly used in conventional high-resolution flat-panel displays into the fine-patterned interconnections,^43,45^ high-resolution QD-LED textile displays can be implemented using a matrix addressing scheme (Figure S12). This approach will effectively overcome the challenges of high-resolution implementation associated with an increasing number of pixels, providing an effective solution to ensure the practicality of high-resolution QD-LED textile displays.

## Conclusion

The investigated ultrathin metal MoO_3_/Au electrode demonstrates superiority over the conventional ITO electrode. Through the combination of 8 nm thick Au layers and 10 nm thick MoO_3_ layer, the electrode exhibits the highest FoM of 0.0077 with a transmittance of *T*_550_ = 81% and a sheet resistance of *R*_s_ = 17.92 Ω sq^–1^. Impressively, the increase in resistance Δ*R*/*R*_0_ remains below 80% after 6000 bending cycles. The MoO_3_/Au/PMA-r structure developed for QD-LEDs exhibits excellent bending durability, maintaining performance for at least 300 cycles at a 5 mm bending radius with no significant loss in luminance. Over 500 cycles, it maintained up to 6325 cd m^–2^ despite a decrease in luminance. Furthermore, the innovative lateral-driving architecture eliminates the need for complex interconnection and reduces the risk of failure of the QD-LED textile display. This breakthrough results in an impressive, fully addressable full-color QD-LED textile display. Woven textile displays utilizing stable and flexible MoO_3_/Au/PMA-r QD-LED structures demonstrate superior robustness and durability. In summary, the realization of a mechanically robust QD-LED textile display has been successfully accomplished. This achievement involves the utilization of cutting-edge ultrathin metal MoO_3_/Au electrodes and the MoO_3_/Au/PMA-r QD-LED structure. The comprehensive development spans material, device, and system levels, providing a stepping-stone for future large-area smart textile displays.

## Supporting Information Available

Testing procedure on a house-made bending test machine; comparison between tensile and compressive stress in bending tests; transmittance of commercial-grade PET/ITO substrates; permanent deformation observed in QD-LED strips after 1000 cycles of bending with a 5 mm radius of curvature; lifetime measurement of MoO_3_/Au/PMA-r device; fabricated MoO_3_/Au electrode for 36-pixel QD-LED strips using a lift-off technique; shift register soldered to the custom PCB for signal transmission to the QD-LED strip; serial driving architecture for the QD-LED textile display; transmittance, sheet resistance, and figure of merit of MoO_3_/Au at various thicknesses (PDF)
