# Supplementary material for: Smart Textile Display with Addressable Quantum Dot Light-Emitting Diode Based on Durable Ultrathin Metal Electrode
Source: ACS Appl Nano Mater. 2025 Mar 18;8(12):5961–70. doi: 10.1021/acsanm.5c00068 (PMC11959519; doi:10.1021/acsanm.5c00068)
Supplement: Supplementary file 1 — an5c00068_si_001.pdf [file an5c00068_si_001.pdf]

Supporting Information

Smart Textile Display with Addressable Quantum Dot Light Emitting Diode based on Durable Ultra-Thin Metal Electrode

Jiajie Yang,[1†] Jeong-Wan Jo,[1†] Yoonwoo Kim,[1] Sung-Min Jung,[1*] Sanghyo Lee,[1,2*] and Jong Min Kim[1]

[1] Electrical Engineering Division, Department of Engineering, University of Cambridge, Cambridge, CB3 0FA, United Kingdom.

[2] School of Materials Science and Engineering, Kumoh National Institute of Technology (KIT), Gumi, 39177, South Korea

[†]J. Yang and J. -W. Jo contributed equally to this work

*Corresponding Author: Prof. Sanghyo Lee and Dr. Sung-Min Jung

Email: sanghyo.lee@kumoh.ac.kr, sj569@cam.ac.uk

This "Supporting Information" includes:

Figure. S1 to S12

Table. S1

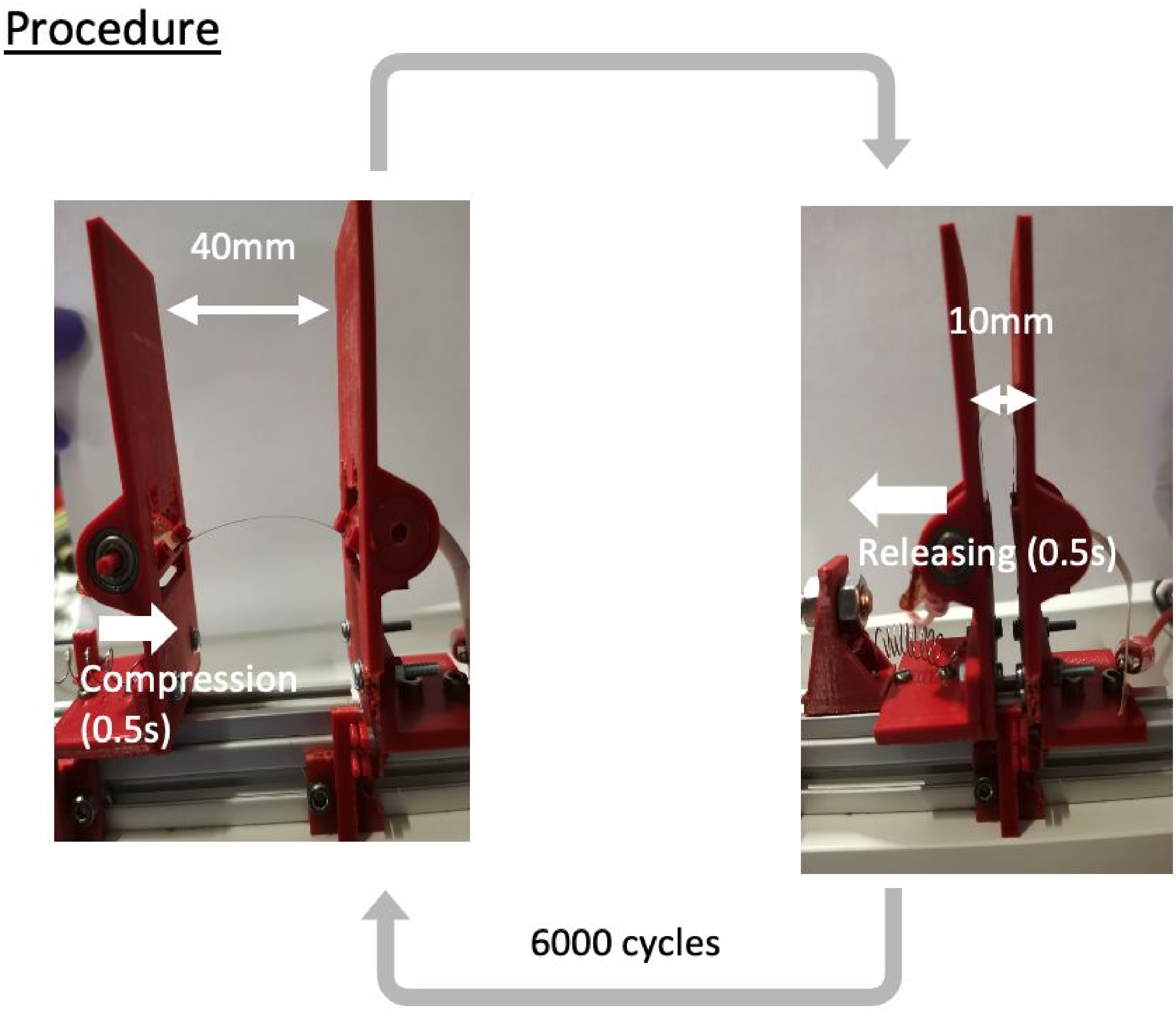

Figure S1. The testing procedure carried out on a house-made bending test machine. The sample is compressed to a desired radius of curvature and then released to a flat state, repeated for 6000 cycles.

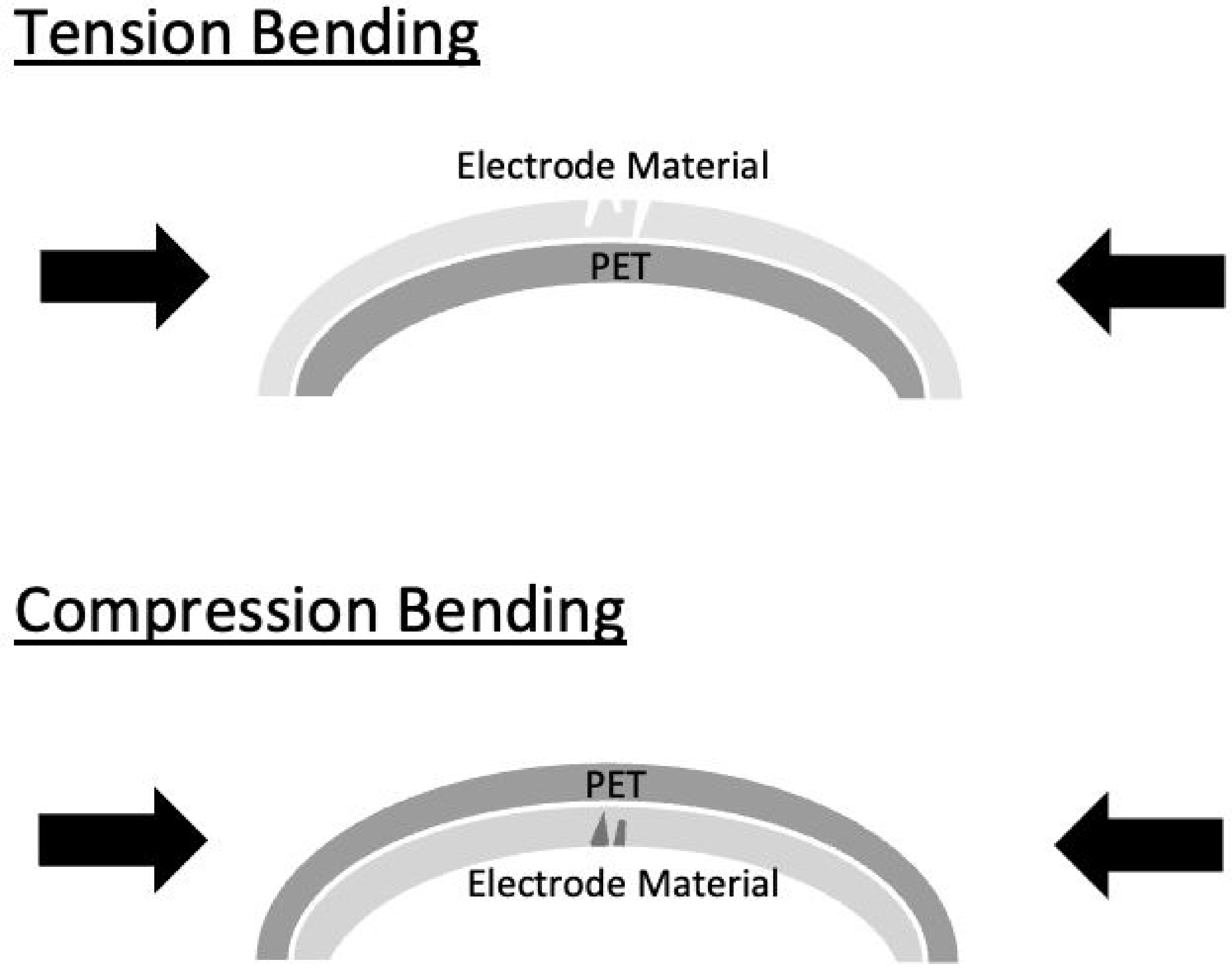

Figure S2. The difference between two types of bending test. In a tension bending, the material to be tested is subjected to tensile stress, whereas in a compression bending, the material is subjected to compressive stress.

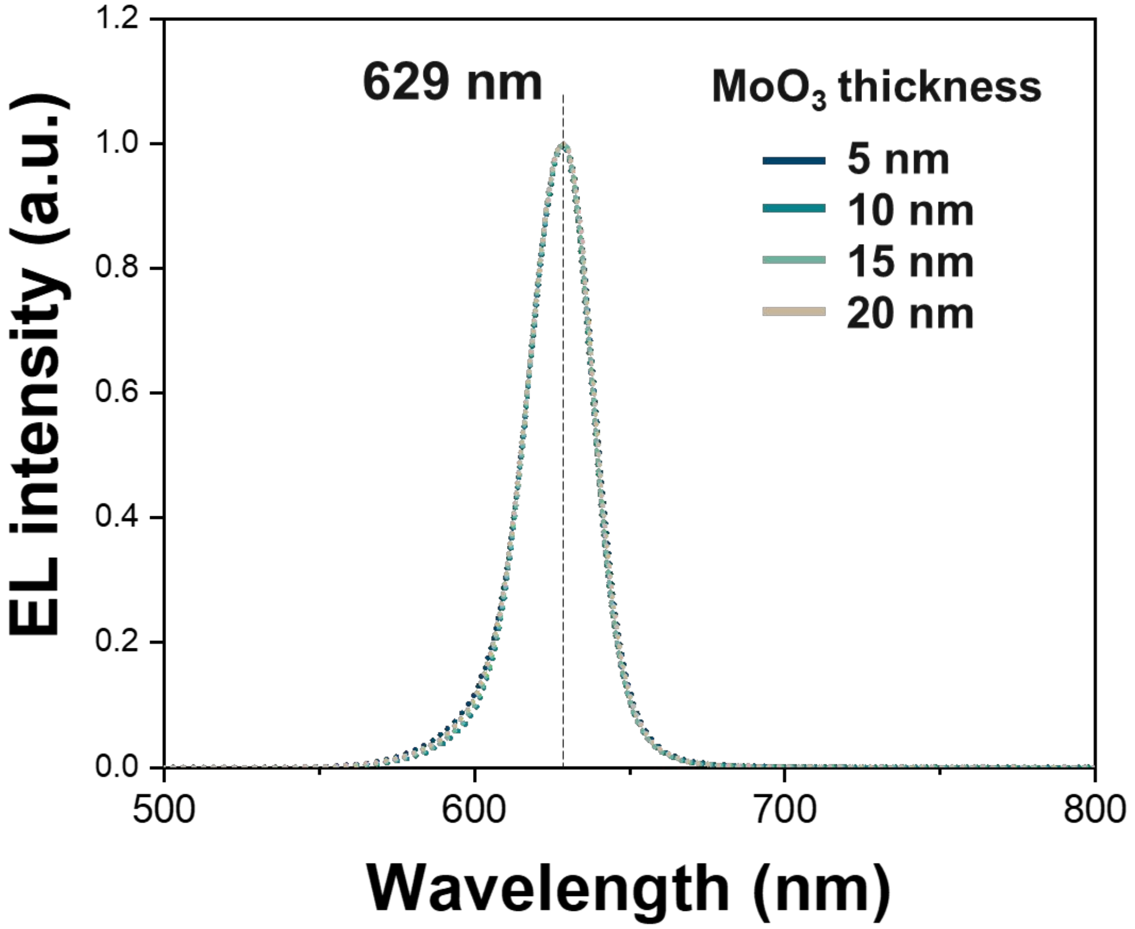

Figure S3. Normalised electroluminescent (EL) spectra of QD-LED devices with MoO_3/Au 8 nm electrodes fabricated with different MoO_3 thicknesses. All devices exhibit a consistent peak wavelength of 629 nm and a full width at half-maximum (FWHM) of 25 nm, indicating that variations in MoO_3 thickness do not significantly affect the EL emission spectra characteristics.

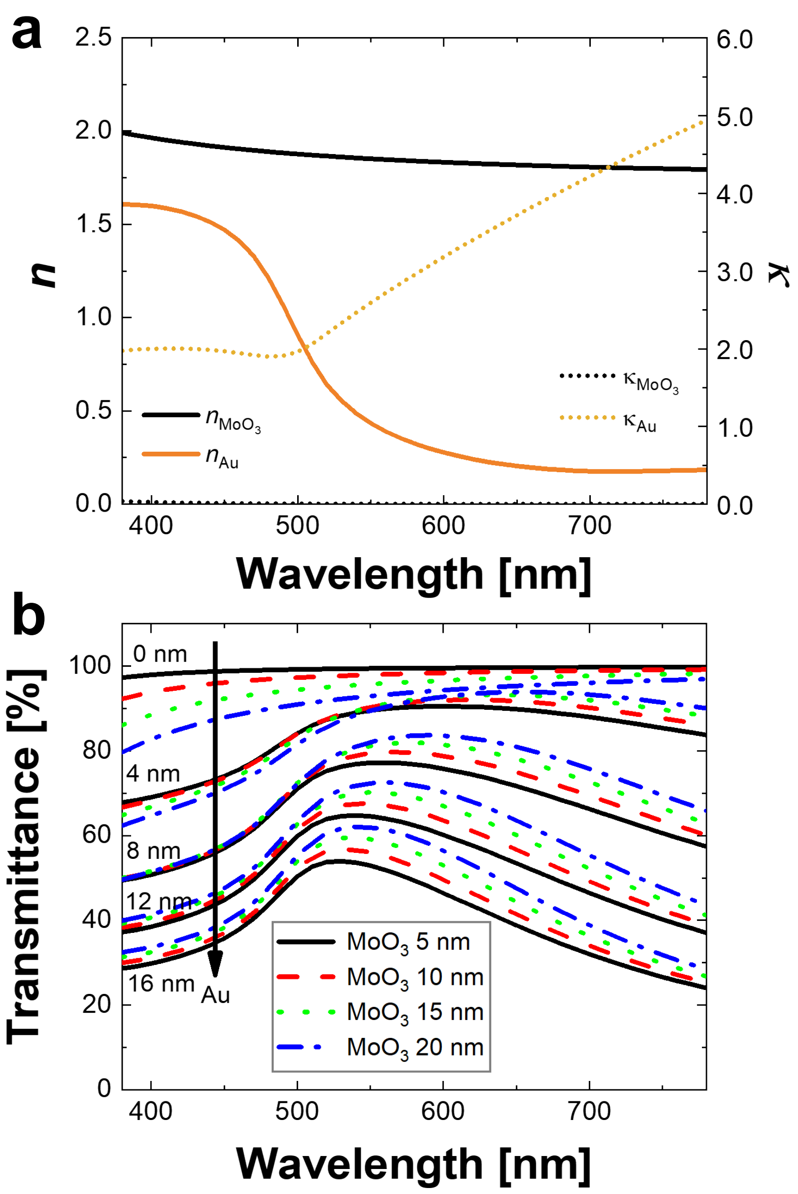

Figure S4. Optical properties of the MoO_3/Au double layer calculated by the transfer matrix method[S1]. (a) Complex refractive indices of MoO_3[S2] and Au[S3] and (b) transmittance for different thickness configurations over the visible range.

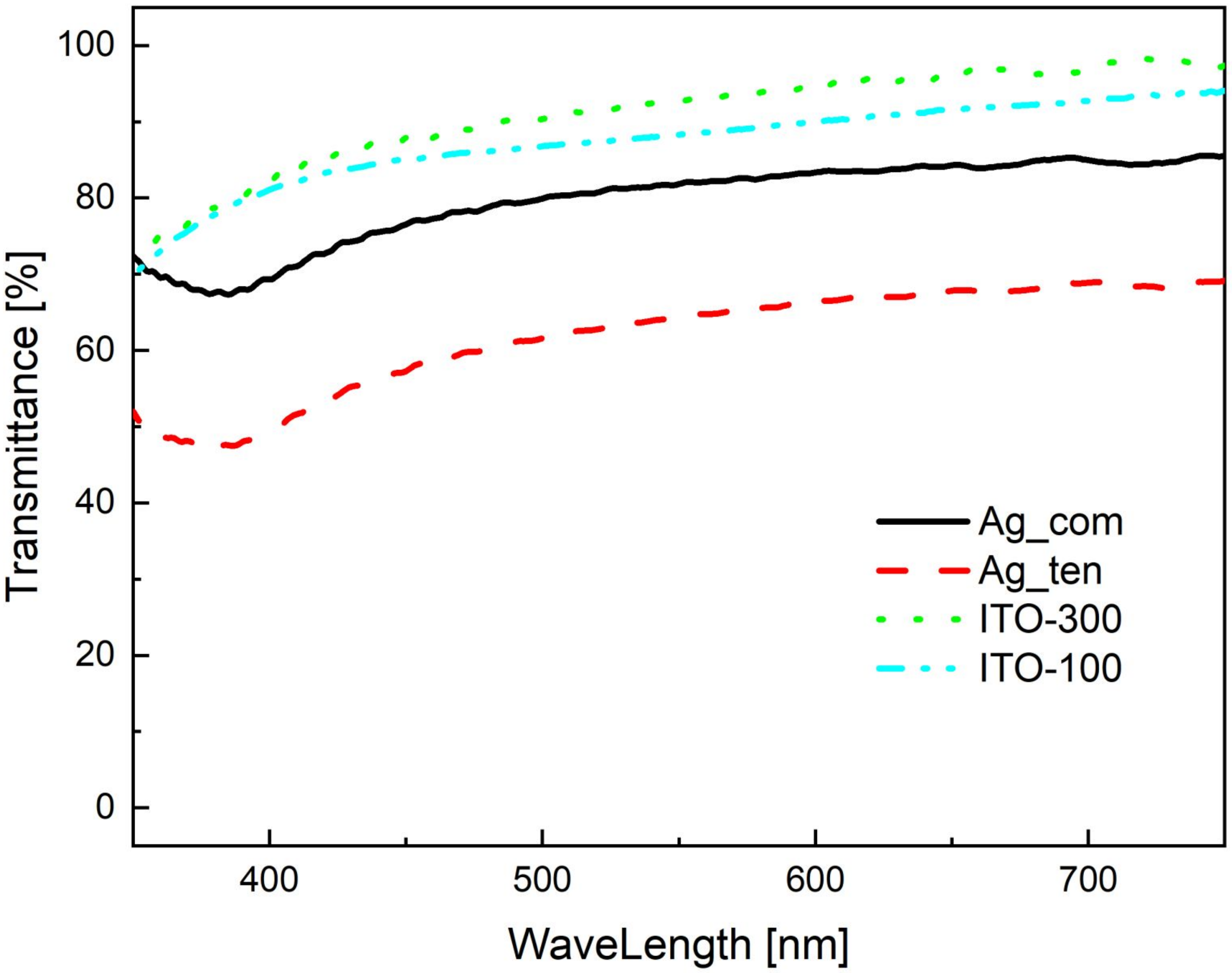

Figure S5. The transmittance of the commercial grade PET/ITO substrate with 100 and 300 Ω sq^{-1} nominal sheet resistance.

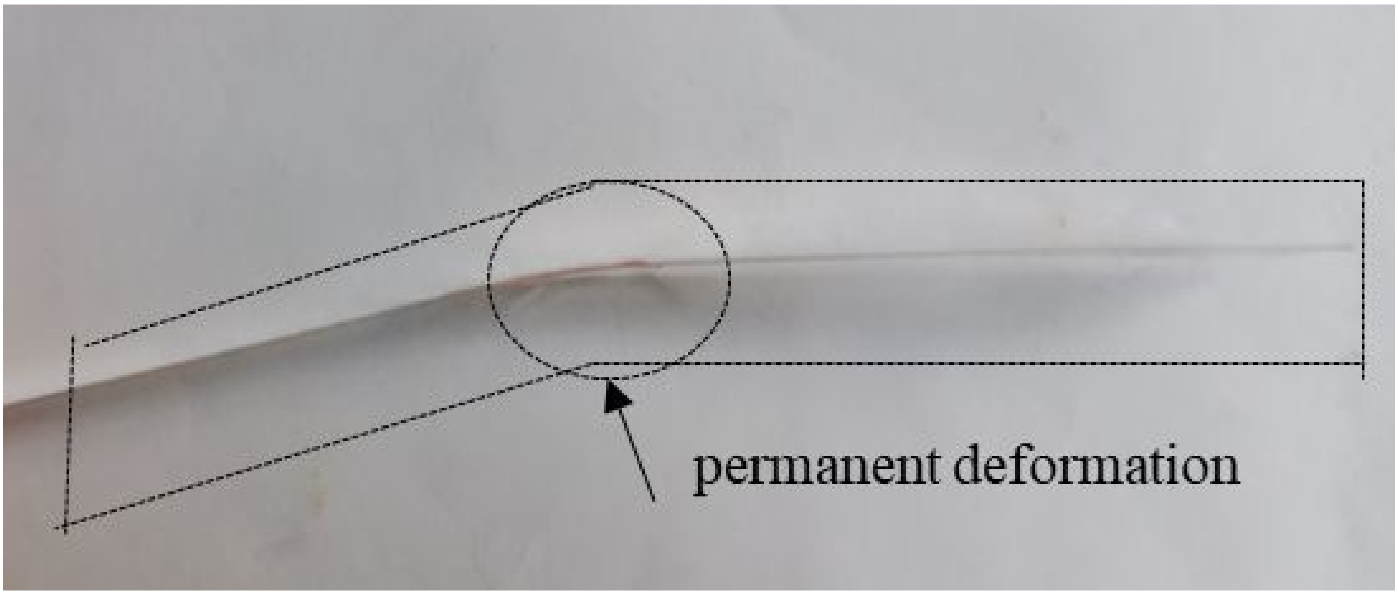

Figure S6. The QD-LED strip undergoing the bending test with a radius of curvature of 5 mm. Permanent deformation of the PET substrate is observed after 1000 bending cycles.

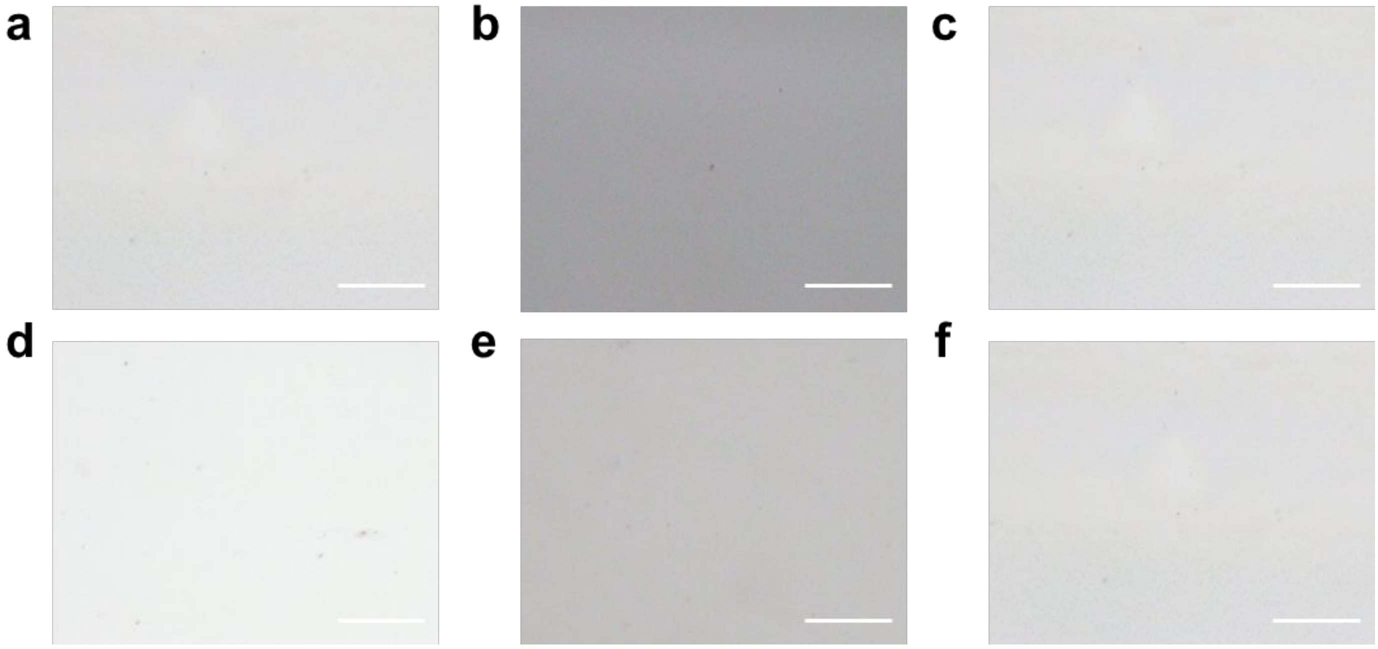

Figure S7. a-c, Optical images of PMA-R (a), red CdSe QD (b), ZnMgO (c) films before bending, **d-f,** Optical images of PMA-R (d), red CdSe QD (e), ZnMgO (f) films after 2000 bending cycles. Scale bar, 50 μm.

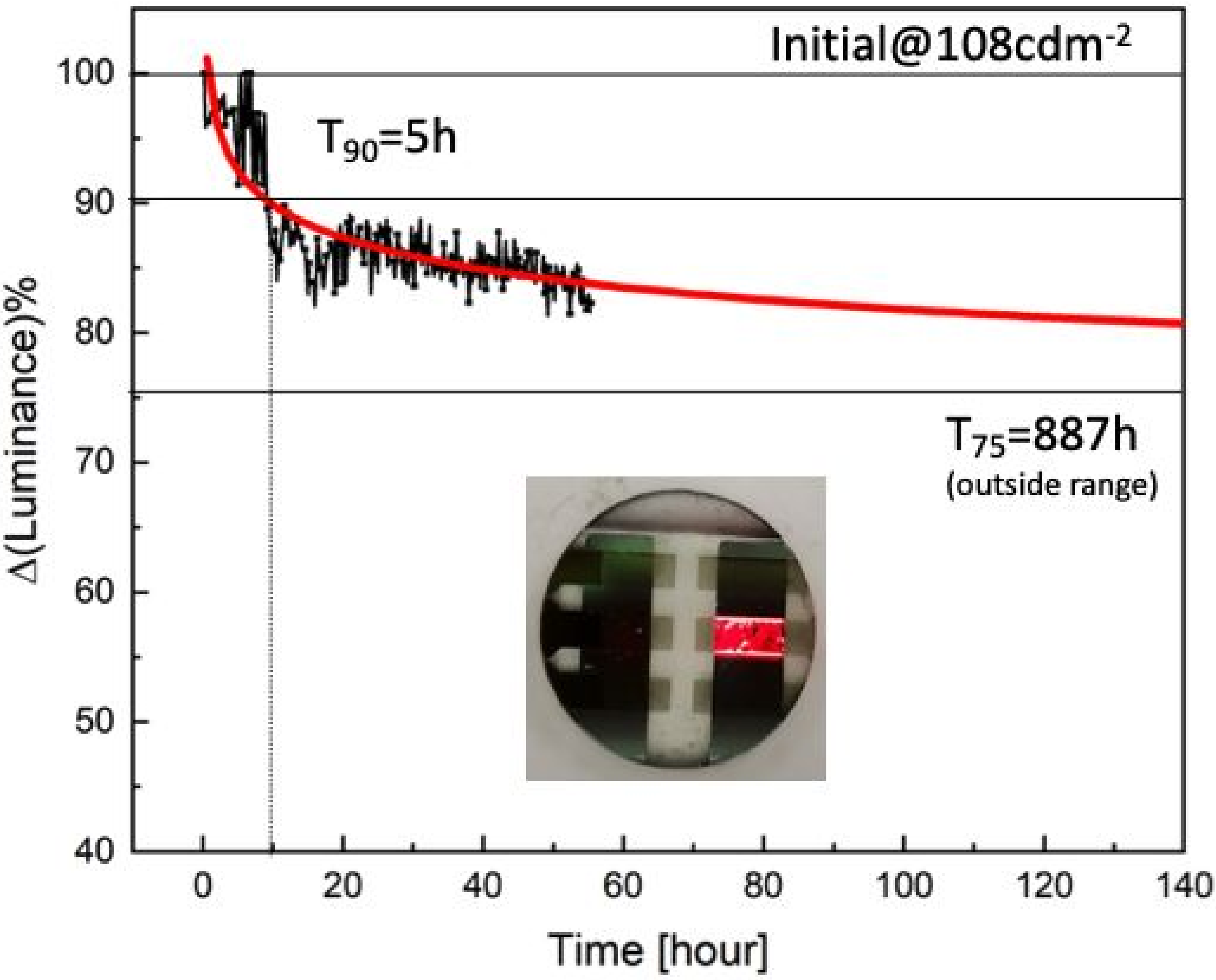

Figure S8. The lifetime measurement of the MoO_3/Au/PMA-r device, showing a lifetime of T_{90} = 5 hours and an excellent T_{75} of over 887 hours, according to the fitted exponential decay curve y = 229×exp (-0.79×0.047).

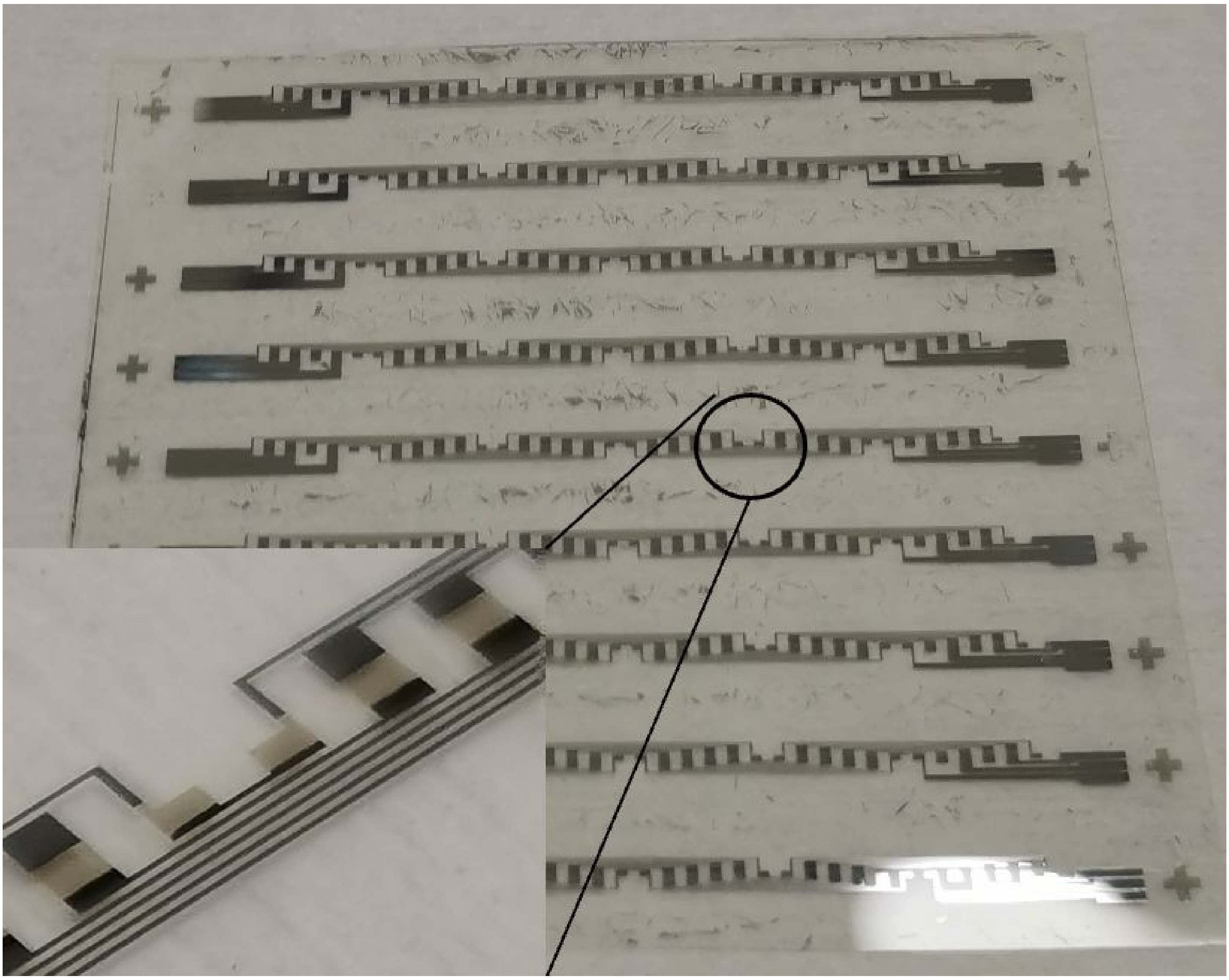

Figure S9. The fabricated MoO_3/Au electrode for 36 pixels QD-LED strips. Multiple strips are fabricated using lift-off technique, demonstrating the scalability of the design.

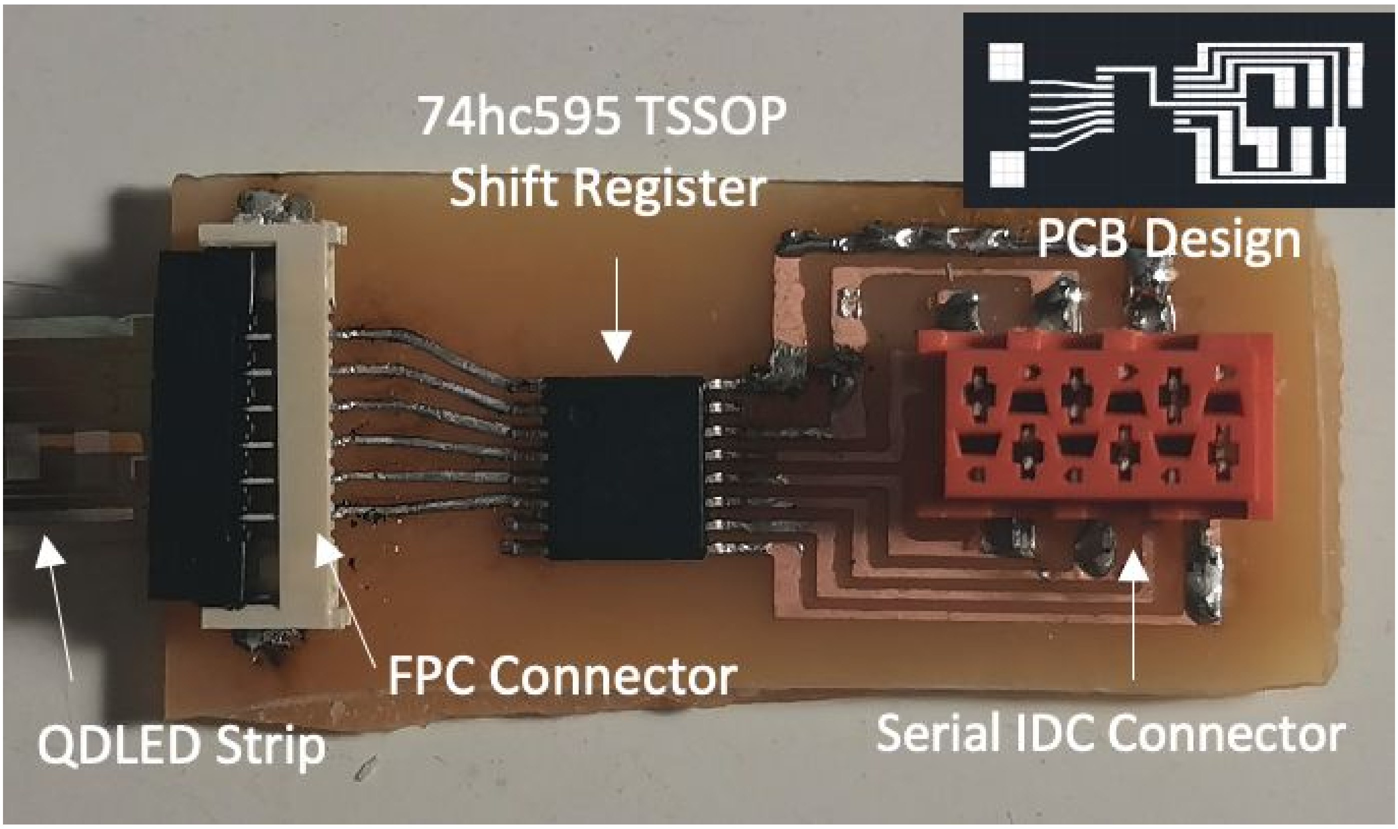

Figure S10. The shift register soldered to the custom designed PCB, attached to each side of the QD-LED strip to transmit the signal.

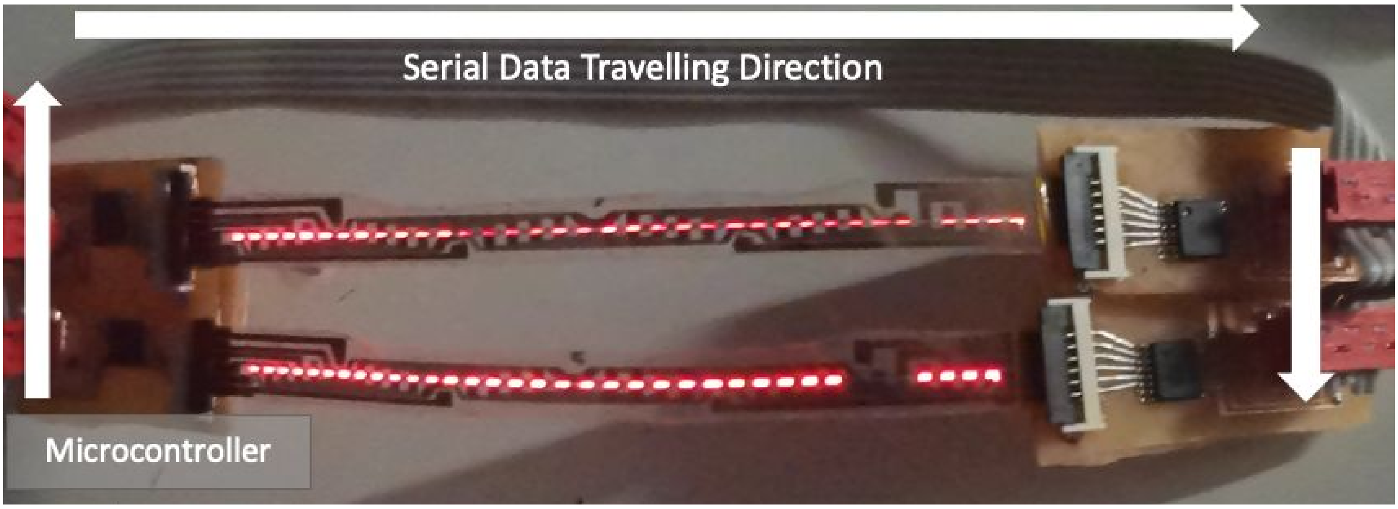

Figure S11. The driving architecture of the QD-LED textile display. Serial data travels from the microcontroller through each strip.

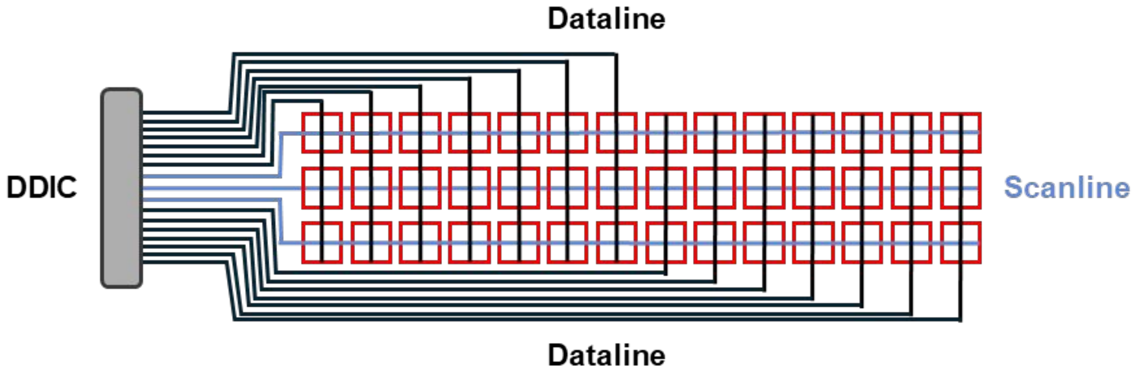

Figure S12. The matrix addressing scheme for high-resolution QD-LED textile displays.

Material	Transmittance at 550 nm T_{550} [%]	Sheet Resistance R_s [Ω/□]	Figure of Merit Φ_H [Ω^{-1}]
Au 4 nm	82.79	301.30	5.03×10^{-4}
Au 8 nm	82.02	17.92	7.69×10^{-3}
Au 12 nm	74.82	8.20	6.71×10^{-3}
Au 16 nm	67.56	6.17	3.21×10^{-3}
ITO 300	92.72	300.00	1.56×10^{-3}
ITO 100	88.26	100.00	2.87×10^{-3}

Table S1. The transmittance, sheet resistance, and figure of merit of MoO_3/Au at various thickness, and together with ITO for comparison. The 8 nm MoO_3 has the best FoM of 0.0077.

References

(S1) Jo, J.-W.; Kim, Y.; Jung, S.-M.; Kim, J. M. Emission-angle-dependent colour properties of full-colour quantum-dot light-emitting diode displays. *Results Phys* 2015, 69, 108130.

(S2) Vos, M.; Macco, B.; Thissen, N. F. W.; Bol, A. A.; Kessels, W. M. M.; Atomic layer deposition of molybdenum oxide from $(NtBu)_2(NMe2)_2Mo$ and O_2 plasma. *J Vac Scia Technol A* 2016, 34, 01A103.

(S3) Yakubovsky, D. I.; Arsenin, A. V.; Stebunov, Y. V.; Fedyanin, D. Yu.; Volkov, V. S. Optical constants and structural properties of thin gold films. *Opt Express* 2017, 25, 25574-25587.
